# Optimisation of xylanases production by two *Cellulomonas* strains and their use for biomass deconstruction

**DOI:** 10.1007/s00253-021-11305-y

**Published:** 2021-05-21

**Authors:** Ornella M Ontañon, Soma Bedő, Silvina Ghio, Mercedes M Garrido, Juliana Topalian, Dóra Jahola, Anikó Fehér, Maria Pia Valacco, Eleonora Campos, Csaba Fehér

**Affiliations:** 1 Instituto de Agrobiotecnología y Biología Molecular (IABIMO), Instituto Nacional de Tecnología Agropecuaria (INTA), Consejo Nacional de Investigaciones Científicas y Técnicas (CONICET), De los Reseros y N. Repetto s/n, Hurlingham, B1686IGC Buenos Aires, Argentina; 2 Biorefinery Research Group, Department of Applied Biotechnology and Food Science, Budapest University of Technology and Economics (BUTE), Szent Gellért tér 4, Budapest, H-1111 Hungary; 3 Centro de Estudios Químicos y Biológicos por Espectrometría de Masa (CEQUIBIEM-FCEN), Departamento de Química Biológica Facultad de Ciencias Exactas y Naturales Universidad de Buenos Aires (UBA-IQUIBICEN), Consejo Nacional de Investigaciones Científicas y Técnicas (CONICET), Buenos Aires, Argentina

**Keywords:** *Cellulomonas*, Lignocellulose, Waste valorisation, Lyophilisation, Enzymatic hydrolysis, Secretome analysis

## Abstract

**Abstract:**

One of the main distinguishing features of bacteria belonging to the *Cellulomonas* genus is their ability to secrete multiple polysaccharide degrading enzymes. However, their application in biomass deconstruction still constitutes a challenge. We addressed the optimisation of the xylanolytic activities in extracellular enzymatic extracts of *Cellulomonas* sp. B6 and *Cellulomonas fimi* B-402 for their subsequent application in lignocellulosic biomass hydrolysis by culture in several substrates. As demonstrated by secretomic profiling, wheat bran and waste paper resulted to be suitable inducers for the secretion of xylanases of *Cellulomonas* sp. B6 and *C. fimi* B-402, respectively. Both strains showed high xylanolytic activity in culture supernatant although *Cellulomonas* sp. B6 was the most efficient xylanolytic strain. Upscaling from flasks to fermentation in a bench scale bioreactor resulted in equivalent production of extracellular xylanolytic enzymatic extracts and freeze drying was a successful method for concentration and conservation of the extracellular enzymes, retaining 80% activity. Moreover, enzymatic cocktails composed of combined extra and intracellular extracts effectively hydrolysed the hemicellulose fraction of extruded barley straw into xylose and xylooligosaccharides.

**Key points:**

*• Secreted xylanase activity of Cellulomonas sp. B6 and C. fimi was maximised.*

*• Biomass-induced extracellular enzymes were identified by proteomic profiling.*

*• Combinations of extra and intracellular extracts were used for barley straw hydrolysis.*

**Supplementary Information:**

The online version contains supplementary material available at 10.1007/s00253-021-11305-y.

## Introduction

Lignocellulosic residues can serve as abundant, cheap, and renewable resources for the production of chemicals, platform molecules, fuels and energy to enhance the development of a sustainable bio-economy (De Bhowmick et al. 2018). Deconstruction of lignocellulosic resources into easily fermentable sugars is very important for the efficient valorisation of biomass. Microbial enzymes with cellulolytic and hemicellulolytic activities have a huge potential to be applied in bioprocesses that use lignocellulosic raw materials (Ballesteros 2010; Maitan-Alfenas et al. 2015).

Decomposition of cellulose requires the concerted action of endo-β-1,4-glucanases (E.C. 3.2.1.4), reducing end-acting cellobiohydrolases (EC 3.2.1.176), exo-β-1,4-glucanases (E.C. 3.2.1.74), cellulose 1,4-β-cellobiosidases (non-reducing end) (E.C. 3.2.1.91) and β-glucosidases (E.C. 3.2.1.21) (Juturu and Wu 2014). However, the hydrolytic deconstruction of hemicellulose requires a higher number of different enzymatic activities due to the variability of its composition and structure (Kane and French 2018). Arabinoxylans are complex hemicelluloses which require a well-balanced action of several enzymes for complete hydrolysis: endo-1,4-β-xylanases (E.C.3.2.1.8), xylan-1,4-β-xylosidases (E.C.3.2.1.37), α-l-arabinofuranosidases (E.C.3.2.1.55), acetylxylan esterases (E.C.3.1.1.72), ferulic/coumaric acid esterases (EC 3.1.1.73) and other specific activities (e.g. α-glucuronidases) depending on the exact chemical composition (Fehér 2018; Bhardwaj et al. 2019).

Efficient enzymatic depolymerisation of the carbohydrate content of lignocellulosic raw material is usually a bottle neck in a biorefinery process. This is why prospection of novel enzymes with higher activity on the raw material used under certain process conditions is required. Moreover, in-site enzyme production is highly recommended for an economically viable operating lignocellulosic biorefinery (Siqueira et al. 2020). Lignocellulosic residues can be used as carbon source for the production of efficient enzyme cocktails within a biorefinery (on-site enzyme production), in order to fulfil or supplement the enzymatic requirements of the process (Astolfi et al. 2019). Cheap and abundant lignocellulose resources, such as wheat bran (Apprich et al. 2014), corn cob (Arumugam and Anandakumar 2016) and paper residues (Wang et al. 2012), have a great potential to be used as the carbon source for biomass degrading enzymes production. The utilisation of lignocellulosic biomass for on-site enzyme production not only can contribute to an efficient waste management, but also offers a sustainable utilisation of these materials adding value to by-products (Das et al. 2013).

In the last decades major research efforts have been made to investigate the production and use of fungal enzymes in biomass conversion. However, there is also a growing interest in the production and application of bacterial enzymes due to their high diversity for several pH and temperature conditions. The fermentation for bacterial enzyme production offers several advantages including high cell growth rate, robustness and low nutritional requirements, resulting in efficient and cheap processes for enzyme production (Maki et al. 2009; Akhtar et al. 2016). Bacteria belonging to the genera *Clostridium*, *Cellulomonas*, *Bacillus*, *Ruminococcus*, *Bacteroides*, *Acetovibrio*, *Streptomyces*, and *Paenibacillus* have been identified as cellulolytic enzyme producers under aerobic or anaerobic culture conditions, depending on the species (Islam 2019). The (hemi)cellulolytic potential of strains belonging to the *Cellulomonas* genus, such as *Cellulomonas fimi* ATCC 484 (Wakarchuk et al. 2016) and *Cellulomonas* sp. B6 (Piccinni et al. 2019) has been demonstrated.

In the present work, we studied the use of different lignocellulosic by-products (wheat bran, corn cob, and waste paper) as carbon sources for *C. fimi* and *Cellulomonas* sp. B6 growth, and also evaluated these feedstocks as inducers of xylanases production and secretion. Additionally, we analysed enzyme production in lab scale bioreactors, studied their potential to deconstruct agro-industrial wastes and evaluated lyophilisation as a method for concentration and conservation of the enzymatic extract.

## Materials and methods

### Microorganisms

*C. fimi* B-402 was obtained from NRRL Agricultural Research Service Culture Collection (IL, USA). *Cellulomonas* sp. B6 was isolated from a bacterial consortium obtained from a preserved native subtropical forest soil sample (Piccinni et al. 2016). Strain *Cellulomonas* sp. B6 has been deposited in public collections DSMZ and NCIMB, as DSM 107934 and NCIMB 15124, respectively. The strains were maintained for long term in Luria-Bertani (LB) medium (10 g/L tryptone, 5 g/L yeast extract, 10 g/L NaCl) (Bertani 1951) with 20% w/w glycerol at – 80 °C. Before use, a loopfull was spread in LB agar plates (LB supplemented by 15 g/L agar) for single colonies isolation.

### Model substrates and biomass feedstocks

Different carbon sources were tested for cell growth and enzyme secretion. Solka-floc (SF, kindly donated by Lund University, Lund, Sweden) and carboxymethyl cellulose of low viscosity (CMC, Sigma-Aldrich, STL, USA) were used as model substrates. The selection of CMC was due to its high purity and solubility in aqueous solutions. SF is a model substrate obtained from milled pinewood through several extraction steps and it has around 76% w/w cellulose and 12% w/w hemicellulose, in terms of dry matter content, and which is insoluble in water (Sipos et al. 2010). Wheat bran (WB), pre-treated sweet corn cob (PSCC) and pre-treated waste paper (PWP) were used as lignocellulosic carbon sources. Sweet corn cob pre-treated by alkali extrusion was provided by INRA (Toulouse, France). Waste paper consisted of corrugated cardboard pieces, which were cut into small pieces (smaller than 0.5 cm), mixed with distilled water to set 10% w/w dry matter content, homogenised with a hand blender for 10 min and autoclaved (121 °C for 30 min). Wheat bran was purchased at a dietary shop.

For enzymatic hydrolysis experiments, extruded barley straw (EBS) was used. EBS was obtained from the Babetreal5 project (https://www.babet-real5.eu/) and provided by CIEMAT (Madrid, Spain). The extrusion conditions were 100 °C, 4.5% NaOH/dry barley straw and neutralisation with H_3_PO_4_ (Duque et al. 2017).

Structural carbohydrates (glucan, xylan and arabinan) and the acid insoluble solids of WB and PWP were determined as described by the National Renewable Energy Laboratory (DEN, USA) (Sluiter et al. 2012). Relative composition of PSCC was provided by INRA. The structural carbohydrate analyses of WB and PWP were carried out in triplicates. Starch content of WB was also determined by using enzymatic treatment with α-amylase as detailed by Bedő et al. (2019). Composition of PSCC, WB and PWP is detailed in Table 1.

**Table 1:** Relative structural carbohydrate content of the lignocellulosic substrates

	Percentage of dry matter		
	PSCC^*^	WB^**^	PWP^**^
Glucan	34.0	34.1 (0.1)	55.8 (0.2)
Xylan	22.6	18.0 (0.2)	13.2 (0.3)
Arabinan	9.3	9.5 (0.4)	1.2 (0.3)
Acid insoluble solid (Klason-lignin)	13.2	7.5 (0.5)	11.5 (0.1)

*PSCC* pre-treated sweet corn cob, *WB* wheat bran, *PWP* pre-treated waste paper

^*^Data provided by INRA

^**^Data determined in this study. Average values and standard deviations (indicated in parentheses) are calculated from triplicates

### Enzyme production in shake flask

Bacteria (*C. fimi* and *Cellulomonas* sp. B6) were grown on minimal medium (MM) (1.67 g/L dipotassium hydrogen phosphate (K_2_HPO_4_), 0.87 g/L potassium dihydrogen phosphate (KH_2_PO_4_), 0.05 g/L sodium chloride (NaCl), 0.1 g/L magnesium sulphate (MgSO_4_×7H_2_O), 0.04 g/L calcium chloride (CaCl_2_), 0.004 g/L iron(III) chloride (FeCl_3_), 0.005 g/L sodium molybdate (NaMoO_4_×2H_2_O), 0.01 g/L biotin, 0.02 g/L nicotinic acid, 0.01 g/L pantothenic acid, 1 g/L ammonium chloride (NH_4_Cl)) supplemented with 1 g/L yeast extract and 1% w/w of different model substrates (SF, CMC) or 1% w/w of different types of biomass as carbon source (WB, PWP or PSCC as indicated). The MM culture media (without microelements) with the substrates was sterilised in autoclave at 121 °C for 20 min, after which microelements (FeCl_3_, NaMoO_4_×2H_2_O, biotin, nicotinic acid and pantothenic acid) were added to the indicated concentration. Starter cultures were obtained by inoculating single colonies (from fresh agar plates) in 10 mL LB medium and incubating at 30 °C and 220 rpm for 24 h, in the case of *Cellulomonas* sp. B6, and for 72 h, in the case of *C. fimi*. Cultures were inoculated from the starter cultures to obtain an initial cell concentration that corresponds to an optical density (OD) of 0.05. Cultures were carried out in 20 mL culture media filled in 100-mL shake flasks, at 30 °C and 220 rpm for 72 h. Cultures were performed in triplicates.

### Enzyme production in bench-top bioreactor

Enzyme production of *C. fimi* and *Cellulomonas* sp. B6 was performed in 500-mL bench-top bioreactors (JFermi Ltd., Jenő, Hungary) filled with 300 mL of MM medium supplemented with 1% w/w PWP and WB, respectively. Bacteria were first grown as starter cultures in LB medium and then inoculated into the MM supplemented with biomass to get an initial cell concentration that corresponds to an OD of 0.05. Enzyme productions, in duplicates, were accomplished at 30 °C for 72 h with daily sampling. The bioreactors were equipped to be able to control dissolved oxygen (DO) level by adjusting the agitation and air flow rate, and to monitor the pH in the culture medium. DO level was kept at 20% of saturation in order to ensure that the culture was maintained in aerobic conditions.

### Production of extracellular enzyme fraction and intracellular enzyme fraction

When the culture of enzyme production reached late exponential phase (72 h), the remaining solid biomass and the obtained cell mass were separated from the supernatant by centrifugation (6000×*g*, 10 min). The supernatants are referred to as extracellular enzyme (EE) fractions. EE fractions were supplemented with 0.04% w/w sodium azide and kept at 4 °C until use. The cells and remaining biomass (centrifugation pellet) were resuspended in citrate buffer (100 mM, pH 6) in a ratio of 1:10 regarding the initial culture volume, ultrasonicated on ice (six pulses of 10 s, 28% amplitude) and centrifuged (10000×*g*, 30 min). After centrifugation, filtered supernatants were used as intracellular enzyme (IE) fractions.

### Lyophilisation of EE and IE fractions

EE and IE fractions were frozen at – 80 °C. Then, they were taken into the freeze-dryer (EDWARDS Super Modulyo Freeze Dryer, Thermo Electron Corp., Waltham, MA, USA), treated at – 30 °C under 1 × 10^−1^ mbar vacuum for 48 h and stored at 4 °C. The lyophilised enzyme fractions were referred to as LEE and LIE in the cases of extracellular and intracellular enzyme fractions, respectively. For enzymatic assays, both fractions were resuspended with sterile water to the original volume. Ten times concentrated enzyme fraction of LEE (LEE (10×)) was also produced by dissolving LEE in appropriate amount of sterile water. Recovery of the xylanase activity was calculated as the ratio between xylanase activity after and before lyophilisation and expressed as a percentage. Lyophilisation experiments were performed in triplicates.

### Enzymatic activity measurements

Xylanase and CMCase activities were assayed in microtubes using beechwood xylan (1% w/w) or CMC (2% w/w) as substrates, respectively (Ghio et al. 2012). For these assays, 0.1 mL of appropriately diluted extracts (EE, IC, LEE, LEE (10×) or LIE) were added to 0.1 mL of each substrate prepared in citrate buffer (pH 6). Hydrolysis reactions were carried out at 40 °C, 400 rpm for 10 min. These conditions were established in a previous work (Piccinni et al. 2019). Reducing sugars released from the reactions were measured by dinitrosalicylic acid (DNS) method (Miller 1959) using glucose or xylose standard curves.

β-Glucosidase, cellobiohydrolase, β-xylosidase and α-l-arabinofuranosidase activities were assayed, using 5 mM *p*-nitrophenyl-β-d-glucopyranoside (*p*NPG), *p*-nitrophenyl-β-d-cellobioside (*p*NPC), *p*-nitrophenyl-β-d-xylopyranoside (*p*NPX) and *p*-nitrophenyl-α-l-arabinofuranoside (*p*NPA) (Sigma-Aldrich, STL, USA) as substrates, according to previously established protocols (Ontañon et al. 2018). In brief, reactions were performed by combining 100 μL of LEE (10×) or LIE fraction with 100 μL of 2.5 mM substrate in citrate buffer (pH 6, 100 mM), and incubating at 40 °C for 20 min. Reactions were stopped with 500 μL of 2% w/w sodium carbonate and absorbance was determined at 410 nm. A *p*-nitrophenol (*p*NP) curve was used as a standard.

All enzymatic assays were conducted in triplicates and controls of enzyme without substrate and substrate without enzyme were included. In all cases, one international unit (U) was defined as the amount of enzyme required to release 1 μmol of product per minute under the assay conditions.

### Analysis of secretome

Secretomic analysis was performed using flasks cultures of *Cellulomonas* sp. B6 in WB and *C. fimi* in PWP, in duplicate according to the protocol previously described by Piccinni et al. (2019). Briefly, total proteins contained in cell-free supernatants were quantified by Bradford assay (Promega, Biodynamics, CABA, Argentina), precipitated with 10% w/w trichloroacetic acid and then resuspended in ultrapure water (resistivity of 18.2 MΩ × cm) to a final concentration of 1 mg/mL. Protein digestion and mass spectrometry analysis were performed at CEQUIBIEM (http://cequibiem.qb.fcen.uba.ar/). Proteins were reduced with dithiotreitol 10 mmol/L for 45 min at 56 °C, alkylated with iodoacetamide (55 mmol/L) for 45 min in the dark and digested with trypsin (PromegaV5111; Promega, Fitchburg, WI, USA) overnight at 37 °C. The digests were analysed by nano LC-MS/MS in a Thermo Scientific Q-Exactive Mass Spectrometer coupled with a nano HPLC EASY-nLC 1000 (ThermoFisher Scientific, Waltham, MA, USA). The MS equipment has a high collision dissociation cell (HCD) for fragmentation and an Orbitrap analyser (ThermoFisher Scientific; Q-Exactive, Waltham, MA, USA). XCALIBUR 3.0.63 (ThermoFisher Scientific, Waltham, MA, USA) software was used for data acquisition and equipment configuration to allow peptides identification at the same time of their chromatographic separation. Full-scan mass spectra were acquired in the Orbitrap analyser. The scanned mass range was 400–2000 *m/z*, at a resolution of 70,000 at 400 *m/z*, and the 12 most intense ions in each cycle were sequentially isolated, fragmented by HCD and measured in the Orbitrap analyser. Peptides with a charge of + 1 or with unassigned charge state were excluded from fragmentation for MS2. Q-Exactive raw data were processed using PROTEOME DISCOVERER software (ver. 2.1.1.21; ThermoFisher Scientific, Waltham, MA, USA) and searched against *Cellulomonas* sp. B6 or *C. fimi* ATCC 484 UniProt sequences database (based on genome full sequences GenBank access LNTD00000000.1 and GCA_000212695.1, respectively), with trypsin specificity and a maximum of one missed cleavage per peptide. Proteome Discoverer searches were performed with a precursor mass tolerance of 10 ppm and product ion tolerance to 0.05 Da. Static modifications were set to carbamidomethylation of Cys, and dynamic modifications were set to oxidation of Met and N-terminal acetylation. Protein hits were filtered for high confidence peptide matches with a maximum protein and peptide false discovery rate of 1% calculated by employing a reverse database strategy. A minimum of two unique peptides was considered as confident detection. For the estimation of relative abundance, we used the protein abundance index emPAI calculated by Sequest using protein identification data. The equation emPAI/Σ (emPAI) × 100 was used to calculate the protein content in mol.% (emPAI%) (Ishihama et al. 2005; Shinoda et al. 2009). The mass spectrometry proteomics data have been deposited to the ProteomeXchange Consortium via the PRIDE partner repository with the dataset identifier PXD022718.

### Enzymatic hydrolysis of EBS and analysis of the hydrolysates

Enzymatic hydrolysis experiments on EBS were performed by using LEE fraction and a combination of LEE and IE fractions with different mixing ratios and dilutions. Enzymatic hydrolysis experiments were carried out in a reaction mixture containing 5% w/v dry EBS in citrate buffer (pH 6, 100 mM). The hydrolysis reactions were carried out at 45 °C, 220 rpm for 72 h and stopped by boiling for 5 min. The supernatant was clarified by centrifugation (6000×*g*, 15 min) and then used to measure the xylose released from the biomass employing d-xylose assay kit (Megazyme, Bray, Ireland). The hydrolysis products were analysed by thin layer chromatography (TLC) in silica gel plates using butanol/acetic acid/water (2:1:1) as solvents and revealed by water/ ethanol/sulfuric acid (20:70:3) with 1% v/v orcinol solution over flame.

## Results

### Xylanase and CMCase activities of EE fractions obtained using different biomass feedstocks as carbon source

**Fig. 1:**
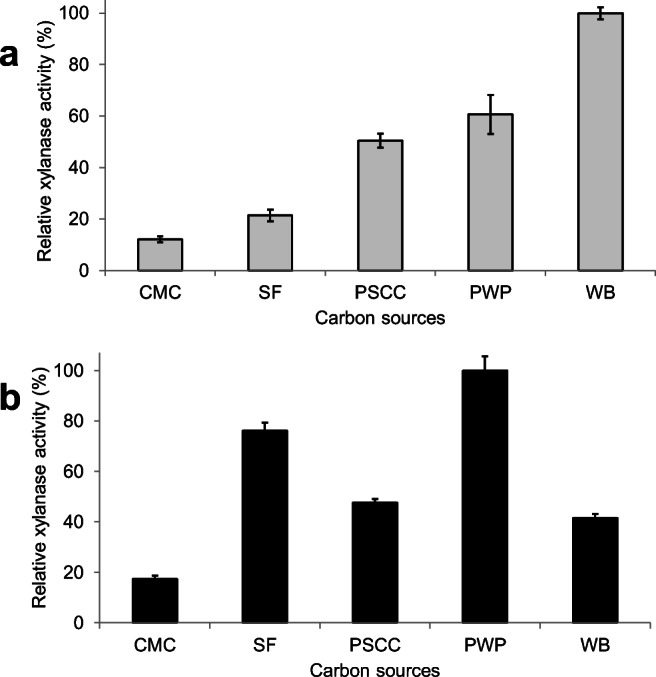
Relative xylanase activities of extracellular enzyme fractions produced by *Cellulomonas* sp. B6 (**a**) and *C. fimi* (**b**) on different carbon sources, from 72 h cultures. CMC: carboxymethyl cellulose, SF: Solka-floc, PSCC: pre-treated sweet corn cob, PWP: pre-treated waste paper, WB: wheat bran. Bars represent average values and standard deviation of triplicate cultures. Relative activities are expressed as the percentage of the maximal activity which was achieved on WB and PWP in the cases of *Cellulomonas* sp. B6 and *C. fimi*, respectively

*C. fimi* and *Cellulomonas* sp. B6 were cultivated on MM supplemented with different lignocellulosic residues as carbon source in order to study the production and secretion of polysaccharide active enzymes (CAZYmes). The carbon sources evaluated in this study were Solka-floc (SF) and carboxymethyl cellulose (CMC) as model cellulosic substrates, and pre-treated waste paper (PWP), wheat bran (WB) and pre-treated sweet corn cob (PSCC) as lignocellulosic materials. The most suitable substrates for culture were selected based on the enzymatic activities measured in the EE fractions. In the case of *Cellulomonas* sp. B6, the highest xylanase activity (3.06 U/mL) was obtained using WB. Xylanase activities produced using PWP and PSCC were 60.6% and 50.5%, respectively, compared to the maximal activity (100%) reached on WB while SF and CMC (model cellulosic substrates) resulted in 21.4% and 12.1% relative xylanase activities, respectively (Fig. 1a).

The highest xylanase activity (1.32 U/mL) of the EE fractions produced by *C. fimi* was achieved by using PWP as the carbon source (Fig. 1b). Surprisingly, the second highest xylanase activity was obtained when SF was used, corresponding to 76.1% activity of the activity achieved on PWP (100%) (Fig. 1b). WB and PSCC resulted in relative xylanase activities of 41.5% and 47.6%, respectively, and the lowest relative xylanase activity (17.4%) was observed with CMC (Fig. 1b). These results suggested that the cellulose-rich, chemically not-modified substrates (SF and PWP) were good inducers for the extracellular production of xylanases by *C. fimi*.

CMCase (glucanase) activities of the EE fractions obtained with the different carbon sources by *C. fimi* and *Cellulomonas* sp. B6 were below 0.2 U/mL in all the cases and were therefore not further studied.

### Enzyme production in bench-top bioreactor

**Fig. 2:**
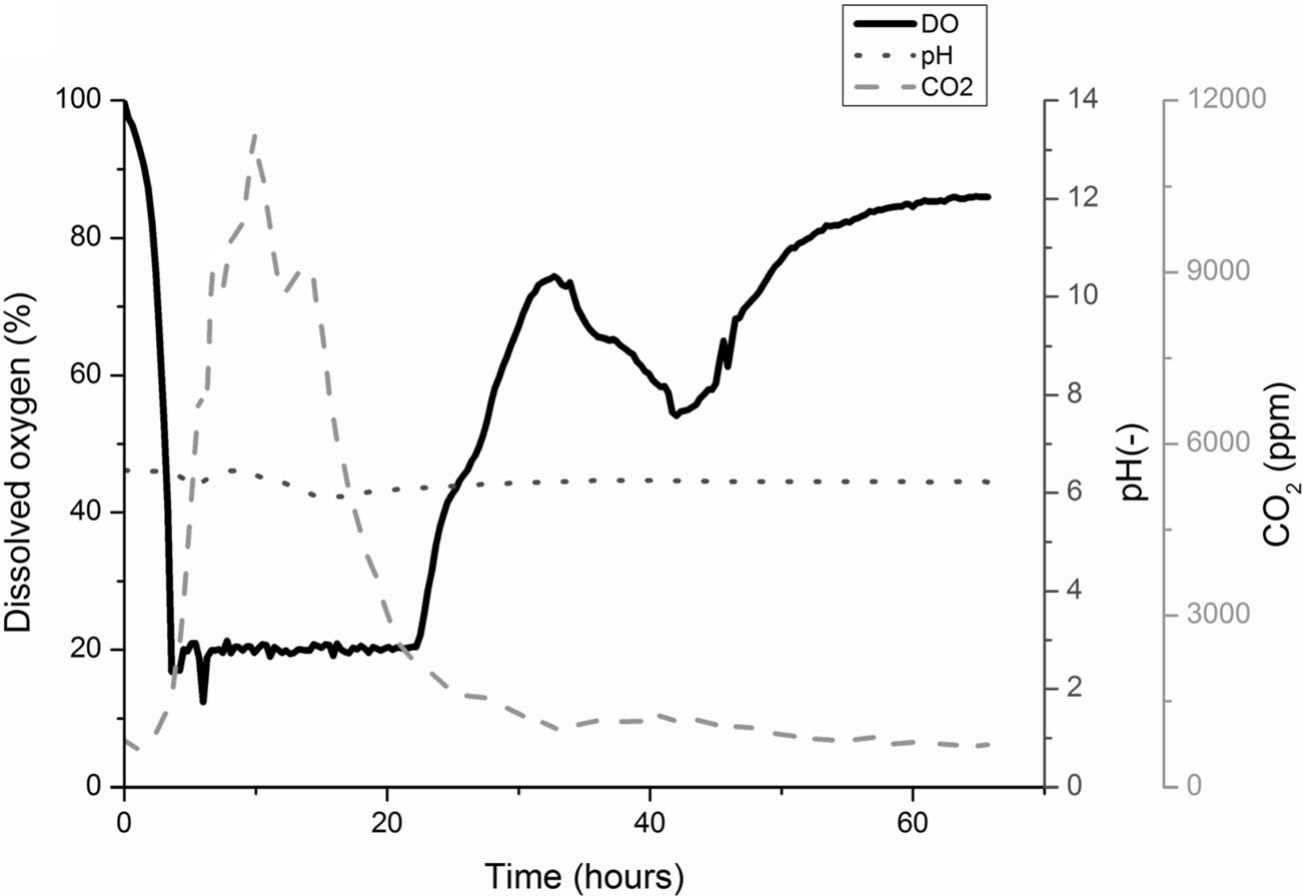
Profile of *Cellulomonas* sp. B6 cultures on wheat bran (WB) in bench-top bioreactor (3L). Temperature and pH were maintained at 30 °C and 7, respectively

Based on the results described in the previous section, WB and PWP were selected as the best carbon sources for extracellular xylanase production by *Cellulomonas* sp. B6 and *C. fimi*, respectively. Hence, scale-up experiments from shake flask to a bench-top bioreactor (300 mL culture volume) were performed only with these raw materials. The culture profile of *Cellulomonas* sp. B6 using 1% w/w WB is shown in Fig. 2. Apart from small deviations in the first 20 h, the pH remained nearly constant (pH 6.8) throughout the experiment; which suggests the absence of pH active by-products, at least in considerable amounts. After 24 h, the DO level started to increase, indicating a decrease of cell growth and/or metabolic activity of the cells. After 48 h, the DO level and CO_2_ concentration in the exhaust gas became nearly constant (Fig. 2).

**Fig. 3:**
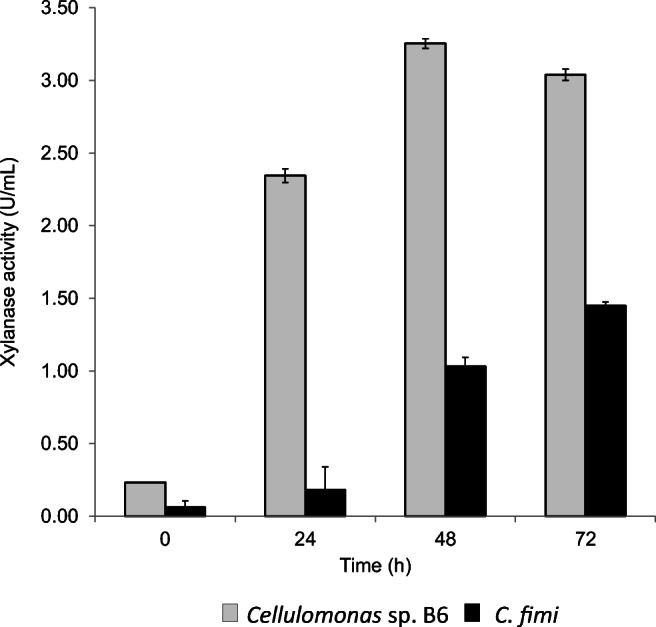
Extracellular xylanase activities during the aerobic culture of *Cellulomonas* sp. B6 (grey bars) and *C. fimi* (black bars) on WB and PWP, respectively, in bench-top bioreactor. WB: wheat bran, PWP: pre-treated waste paper

The xylanase activity displayed a significant increase after 24 h and reached the highest activity at 48 h for *Cellulomonas* sp. B6 and at 72 h for *C. fimi* (Fig. 3). Under these conditions, the extracellular xylanase activity of *C. fimi* was lower than that of *Cellulomonas* sp. B6, which is consistent with the results obtained in flasks.

The extracellular xylanase activity for *Cellulomonas* sp. B6 grown in bench-top bioreactor (~ 3 U/mL) was similar to that obtained when grown in flasks (3.06 U/mL), after 72 h of fermentation using WB. For *C. fimi*, the outcome was similar: similar xylanase activities either grown in shake flask or bioreactor using PWP (~ 1.4 U/mL). These results prove the scalability of the process. After 2 days of fermentation, the xylanase activity in the EE fraction of *Cellulomonas* sp. B6 was three times higher than that of in the EE fraction of *C. fimi*.

### Secretome analysis

With the aim to correlate the observed xylanase activity with the specific secreted proteins, extracellular extracts (EE) were analysed by mass spectrometry, in duplicate independent cultures for each strain. In the WB extracellular fraction of *Cellulomonas* sp. B6, 197 proteins were detected in both samples. In the case of *C. fimi* PWP extracellular fraction, 360 total proteins were identified in both samples. From all of these proteins, 28 and 25 corresponded to glycoside hydrolases (GH) in *Cellulomonas* sp. B6 and *C. fimi*, respectively (Table 2). *Cellulomonas* sp. B6 secreted a repertoire of xylansases (7 GH10 and 1 GH11), which could account for the high xylanase activity in the culture supernatant. In particular, 4 of these xylanases (3 GH10 and 1 GH11) were amongst the most abundant proteins in the extract (from 0.77 to 10% of total proteins secreted), based on the index of relative abundance (emPAI%). The most abundant putative CAZymes in the WB secretome were a GH62 α-l-arabinofuranosidase (a debranching enzyme), two GH10 xylanases, a GH6 exo-glucanase and a GH9 endoglucanse. As in previous studies, a high abundance of extracellular components of putative ABC transporters were also identified (Piccinni et al. 2019). By culture in PWP, *C. fimi* secreted 4 GH10 (out of the 5 GH10 encoded in the genome) and 1 GH11 (the only one predicted). The most abundant protein was a xylanase (UNIPROT id F4H4N7) which represented around 15% of total proteins, followed by a GH9 endoglucanase (about 3%) and three other xylanases (0.5 to 1.5% of total proteins) (Table 2).

**Table 2:** Glycoside hydrolases (GH) identified in the culture supernatants of *Cellulomonas* sp. B6 and *Cellulomonas fimi* grown on wheat bran (WB) and pre-treated waste paper (PWP), respectively, (72 h, duplicate cultures). Proteins were identified by mass spectrometry and the relative abundance index (emPAI%) was calculated in each sample (named as 1 and 2) by Sequest using protein identification data

Accession UNIPROT	CAZY domains and protein probable function description	emPAI%	
		**B6 (WB)1**	**B6 (WB)2**
A0A0V8TB53	GH10 β-xylanase	9.67	3.76
A0A0V8TA57	GH9-CBM4 endo-glucanase	8.67	4.98
A0A0V8S5R6	GH6 exo-glucanase	7.47	2.00
A0A0V8SBR8	GH62-CBM13 α-l-arabinofuranosidase	5.58	14.13
A0A0V8SJU5	GH10-CBM2 β-xylanase	4.81	3.76
A0A0V8SMW4	GH10-CBM13 β-xylanase	2.02	0.85
A0A0V8TC27	GH6-CBM2 endo-glucanase	1.35	0.66
A0A0V8ST89	GH10-CBM9 β-xylanase	1.10	1.22
A0A0V8SFL7	GH11-CBM2 endo-1,4-β-xylanase	0.77	1.77
A0A0V8S6F9	GH48-CBM2 exoglucanase	0.62	0.29
A0A0V8T9P9	GH9 endoglucanase	0.58	0.49
A0A0V8SE95	GH18-CBM2 chitinase	0.56	0.56
A0A0V8T9W1	GH10 xylanase	0.37	0.61
A0A0V8T944	GH9-CBM2 endoglucanase	0.36	0.36
A0A0V8S2E5	GH5 endoglucanase	0.32	0.70
A0A0V8SBG4	GH16-CBM13 1,3-β-glucanase	0.29	0.29
A0A0V8T8K7	GH5 β-mannosidase	0.28	0.46
A0A0V8TC25	GH27-CBM13 α-galactosidase	0.18	0.18
A0A0V8SMY7	GH43-CBM13 β-xylosidase	0.14	0.19
A0A0V8TAG7	GH74 xyloglucanase	0.13	0.10
A0A0V8S7T2	GH10 β-xylanase	0.12	0.18
A0A0V8S3C7	GH10 β-xylanase	0.10	0.26
A0A0V8TBY6	GH51 α-l-arabinofuranosidase	0.09	0.10
A0A0V8S1S2	GH51 α-l-arabinofuranosidase	0.08	0.07
A0A0V8SI61	GH3 β-glucosidase	0.09	0.08
A0A0V8T8K6	GH3 β-glucosidase	0.06	0.04
A0A0V8TBD5	AA10 LPMO	0.05	0.14
A0A0V8TBM3	GH43-CBM13 β-xylosidase	0.05	0.06
		***C. fimi*** **(PWP)1**	***C. fimi*** **(PWP)2**
F4H4N7	GH10-CBM2 β-xylanase	14.50	15.89
P14090	GH9 endoglucanase	2.46	3.96
F4H710	GH11 endo-1,4-β-xylanase	1.12	1.67
F4H454	GH10-CBM2 β-xylanase	0.74	1.07
F4GZV4	GH10-CBM13 β-xylanase	0.49	0.25
P50899	GH48-CBM2 exo-glucanase	0.31	0.36
F4H413	GH6 endoglucanase	0.27	0.16
F4H2N5	GH13 α-1,6-glucosidase	0.26	0.30
F4H3U7	GH9 endoglucanase	0.25	ND
F4H0M7	GH26-CBM23 endo-1,4-β-mannosidase	0.19	0.17
F4GZL0	GH5-CBM2 endoglucanase	0.09	0.13
F4GZY2	GH6-CBM2 endoglucacanase	0.12	0.07
F4GY46	GH10 β-xylanase	0.12	0.11
F4GY55	GH3-CBM11 β-glucosidase	0.07	0.07
P50401	GH6-CBM2 exoglucanase	0.07	0.05
F4H4T8	GH3 β-glucosidase	0.03	0.02
F4GZV3	GH62-CBM13 α-l-arabinofuranosidase	0.03	0.02
F4H0T4	GH3 β-glucosidase	0.01	0.02
F4H120	GH3 β-glucosidase	0.03	ND
F4H5F6	GH81-CBM16 endo-1,3(4)-β-glucanase	0.02	ND
F4H166	GH51 α-l-arabinofuranosidase	0.01	0.01
F4GZJ9	GH74-CBM2 xyloglucanase	0.01	0.01
F4H7D2	GH36 α-galactosidase	0.01	0.01
F4H174	GH64-CBM13 endo-1,3-β-d-glucosidase	ND	0.01
F4H7E7	GH31 α-glucosidase	0.01	0.01
F4H0J4	GH51 α-l-arabinofuranosidase	0.01	ND

*ND* not detected

### Activities of lyophilised extracts of *Cellulomonas* sp. B6

As *Cellulomonas* sp. B6 presented the highest xylanase activity in the secreted fraction, and this activity correlated with a higher number of xylanases identified in the secretome, this strain was chosen for further studies. Lyophilisation of the enzymatic extracts (EE and IE fractions) produced by *Cellulomonas* sp. B6 growing on WB was analysed in order to provide an efficient method to preserve enzymatic activities and concentrate the fractions. Lyophilisation of EE fraction (LEE) of *Cellulomonas* sp. B6 resulted in 80% recovery of xylanase activity and could be concentrated 10 times (LEE (10×)). The IE fraction was not further concentrated after lyophilisation (LIE). Beyond the main xylanase activity, β-xylosidase, α-arabinofuranosidase, CMCase, β-glucosidase and cellobiohydrolase activities of LEE (10×) and LIE fractions were also determined (Table 3). While xylanase activity was more than 10 times lower in LIE fraction compared to that obtained in LEE (10×), additional activities relevant to hemicelluloses deconstruction, such as α-l-arabinofuranosidases and β-xylosidases were similar in both fractions (Table 3).

**Table 3:** Activities of the lyophilised extracellular and intracellular enzyme fractions of *Cellulomonas* sp. B6 grown on WB. LEE (10×): 10 times concentrated extracellular enzyme obtained from lyophilised sample

Activity (U/mL)		
	LEE (10×)	LIE
Xylanase	32 (5.1)	1.9 (0.8)
CMCase	3.6 (0.5)	0.5 (0.2)
α-arabinofuranosidase	1 (0.3)	1.1 (0.3)
β-xylosidase	0.4 (0.1)	0.5 (0.0)
β-glucosidase	< 0.1	< 0.1
Cellobiohydrolase	0.1	< 0.1

*LIE* lyophilised intracellular enzyme, *CMCase* carboxymethyl cellulase, *WB* wheat bran. Average values and standard deviations (in parentheses) were calculated from biological triplicates

### Enzymatic hydrolysis of EBS by *Cellulomonas* sp. B6 enzymatic extracts

Enzymatic hydrolysis of EBS using the xylanolytic extracts of *Cellulomonas* sp. B6 grown on WB was performed to test their hydrolytic efficiency and applicability in lignocellulosic bioprocesses. For this purpose, LEE alone or in combination with IE were used. As no concentration was required in the case of the intracellular extract, the fresh fraction of IE (before lyophilisation) was used, which had shown the same enzymatic activity as LIE. The different enzyme mixtures tested in enzymatic hydrolysis of EBS contained LEE and IE fractions with the following xylanase activity: 30 U/mL of LEE, 15 U/mL of LEE, 7.5 U/mL of LEE, 15 U/mL of LEE with 1.5 U/mL of IE, 7.5 U/mL of LEE with 0.75 U/mL of IE, and 3 U/mL of LEE with 0.3 U/mL of IE. Control experiments without the addition of enzyme extracts were also performed.

**Fig. 4:**
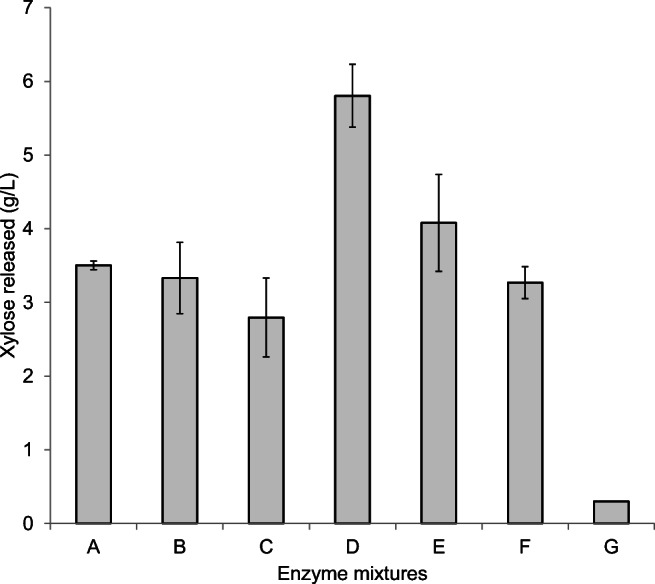
Released xylose during hydrolysis of extruded barley straw (EBS) by using different mixtures of extracellular (LEE) and intracellular (IE) fractions of *Cellulomonas* sp. B6 grown on wheat bran (WB). The enzyme mixtures were the following: 30 U_xylanase_/mL of LEE (**A**), 15 U_xylanase_/mL of LEE (**B**), 7.5 U_xylanase_/mL of LEE (**C**), 15 U_xylanase_/mL of LEE + 1.5 U_xylanase_/mL of IE (**D**), 7.5 U_xylanase_/mL of LEE + 0.75 U_xylanase_/mL of IE (**E**), and 3 U_xylanase_/mL of LEE + 0.3 U/mL xylanase of IE (**F**).  G: Substrate control without enzymes. Xylose concentration was determined by enzymatic colorimetric assay from d-xylose Kit (Megazyme)

In all tested conditions, a xylose concentration higher than 2.5 g/L was released from EBS (Fig. 4). Supplementation of the LEE fraction with IE fraction increased the amount of xylose released from biomass from 3.3 to 5.8 g/L, when 15 U_xylanase_/mL of LEE supplemented with 1.5 U_xylanase_/mL of IE (Fig. 4B, D) and from 2.8 to 4.1 g/L when 7.5 U_xylanase_/mL of LEE was supplemented with 0.75 U_xylanase_/mL of IE (Fig. 4C, E), probably due to additional activities, involved in hemicellulose degradation, present in the IE. This result was further confirmed by performing thin layer chromatography (TLC) analyses of the hydrolysates of EBS, observing an increase in the conversion of oligosaccharides to xylose in enzyme mixtures containing IE. A significant amount of short chain soluble oligosaccharides were also produced (Supplementary Fig. S1). Hence, supplementing the extracellular enzymatic extracts (LEE) with the intracellular (IE) fraction significantly enhanced the liberation of xylose from EBS.

## Discussion

In the current work, we studied the feasibility of using different carbon sources—purified polysaccharides and cheap agro-industrial residues—as selective inducers of (hemi)cellulolytic activity in *C. fimi* B-402 and *Cellulomonas* sp. B6, already known as lignocellulose-active bacteria. Altogether, both strains showed greater xylanolytic than cellulolytic activity. While *Cellulomonas* sp. B6 showed higher activity on lignocellulosic complex biomass, cellulose-rich (chemically not-modified) substrates (SF and PWP) were better inducers for the extracellular production of xylanases by *C. fimi.* In addition, although the growth on complex biomass has served as a successful strategy to enhance xylanolytic activity in several microorganisms (Saratale et al. 2010; Takenaka et al. 2019), it has also been proven that cellulosic substrates can induce the production of xylanases in *C. fimi* (Wakarchuk et al. 2016). The use of residues and by-products as substrates for microbial growth has the advantage of being an inexpensive strategy for inducing the production of enzymes (Lopes et al. 2018). Interestingly, we found that low-value substrates of wheat bran (WB) and pre-treated waste paper (PWP) were the best inducers of xylanase activity in *Cellulomonas* sp. B6 and *C. fimi*, respectively. Proteomic analysis revealed that all the extracellular xylanases (GH10, GH11) and cellulose active enzymes (GH6, GH9, GH48) encoded in *Cellulomonas* sp. B6 genome were secreted by growth on WB. These findings, along with previously reported assay, confirm that complex substrates, such as WB, are highly suitable for the production of CAZYmes, especially those active on hemicellulose, by *Cellulomonas* sp. B6 (Piccinni et al. 2019). Eleven enzymes belonging to families GH6, GH48, GH9, GH10 and GH11, commonly associated with endo-xylanase and endoglucanase activity, were also secreted by *C. fimi* growing on PWP. By contrast, other substrates like CMC, WB or xylan have been reported to induce the secretion of only some of these enzymes in the model strain *C. fimi* ATCC 484 (Wakarchuk et al. 2016; Spertino et al. 2018). Moreover, as described by Wakarchuk et al. (2016) the genome of *C. fimi* ATCC 484 encodes 5 GH10 and 1 GH11 predicted xylanases. In the current study, PWP induced the secretion of 4 GH10 and 1 GH11, which is the maximum amount of secreted xylanases reported for *C. fimi* to date.

All of the above-mentioned carbohydrate-active enzymes have a broad range of industrial applications. Therefore, their production, catalytic efficiency and activity-preservation are essential factors from a biotechnological perspective. Bench-scale bioreactors have resulted to be useful tools for the identification and validation of key parameters involved in the scaling-up of bioprocesses (Marques et al. 2010). In the current study, the xylanase activity of both strains growing in a stirred tank bioreactor employing their preferred substrates was comparable with the one obtained in shake flask cultures. Conversely, some researchers have reported a significant yield loss by transferring the enzyme fermentation to bench-scale bioreactor (Kumar et al. 2009; Garai and Kumar 2013). Oxygen limitation, reduced availability of insoluble substrate, shear induced by agitation, dilution, substrate and product concentration are amongst the main listed drawbacks (Yegin et al. 2017). Remarkably, *Cellulomonas* sp. B6 and *C. fimi* showed a good performance in bench-scale bioreactors, foreseeing a great chance of efficient enzyme production on an industrial scale. It is important to note that 1% w/w PWP resulted in a dense and slimy suspension, which was a difficult medium to work with, especially because it can easily cause inhomogeneity in the bioreactor. The xylanase activity in the culture broth of *C. fimi* continuously increased during the fermentation. Thus, longer fermentation times should be examined to determine the maximum xylanase activity that may be achieved by *C. fimi* grown on PWP in a bioreactor. However, increasing fermentation time also increases the production costs. Process optimisation, including different agitation rates, addition of antifoam or modifications in substrate concentration, could be accompanied by improved performance and reduced operating times. The profiles of CO_2_ level in exhaust gas, DO concentration and xylanase activity in the culture medium suggest that the xylanase production by *Cellulomonas* sp. B6 on WB might have a growth-associated characteristic. A similar trend was observed by Xu et al. (2005) when the production of xylanase extract by *Pseudomonas* sp. WLUN024 was studied.

Concentration of enzymes is an important step in their production, especially if it helps to overcome the need for preservation and transportation at low temperatures. Many commercial enzyme preparations consist of concentrated culture broth with additives that stabilise enzymatic activity (Poletto et al. 2015). In the current work, the extracellular extracts from *Cellulomonas* sp. B6 retained 80% of xylanase activity after lyophilisation and further re-suspension, without the need of stabilising additives. This is a promising result since freeze-drying is a simple method for enzyme preservation but is usually associated with protein denaturation (Berghout et al. 2015). Analysis of different glycoside hydrolase activities in the lyophilised samples showed that the extracellular enzyme fraction contained activities for polymeric hydrolysis in much higher quantities than activities for side-chain sugar hydrolysis. However, side activities were abundant in the intracellular enzyme fraction. Similarly, Rajoka (2005) reported that the cell-free supernatants of *Cellulomonas flavigena* NIAB 441 culture exhibited greater endo-xylanase activity than cell-associated β-xylosidase activity.

Lyophilisation allowed us to concentrate the xylanase activity up to 10-fold and to use the extracts for hydrolysis of extruded barley straw (EBS). In order to improve the process, we supplemented the extracellular extract with the intracellular enzymes released by sonication. Using the enzyme cocktails containing different amount of LEE and IE fractions of *Cellulomonas* sp. B6 for enzymatic hydrolysis experiments of EBS resulted in the release of xylose and xylooligosaccharides as the main soluble products. Supplementing LEE samples with appropriate amounts of IE fraction significantly enhanced xylose release from EBS. Addition of appropriate xylanases during the enzymatic deconstruction of pre-treated barley straw is of great importance, since xylanase supplementation can significantly improve the efficiency of cellulose-hydrolysis (García-Aparicio et al. 2007). Moreover, hydrolysis by xylanases alone can result in promising bio-products like prebiotic xylo-oligomers or monomer xylose which may be then converted into high-value products (e.g. xylitol). Hence, LEE and IE from *Cellulomonas* sp. B6 may be interesting biotechnological candidates for biomass hydrolysis to produce xylo-oligomers, xylose or enhance enzymatic cellulose deconstruction.

We have concluded that the xylanolytic activity of *Cellulomonas* sp. B6 and *C. fimi* was differentially induced by varying the substrates utilised for its growth. Low-cost lignocellulosic residues were the most suitable inducers of secreted hemicellulases, which were then efficient for biomass decomposition into xylose and xylooligosaccharides. Fermentation in a bench-scale bioreactor followed by supernatant lyophilisation was a successful tool to increase enzyme production and concentrate enzymatic activity. The larger scale-up parameterisation and the design of xylanolytic cocktails are some of the further objectives that emerge from this research. Thorough study of the molecular mechanisms involved in the activity could also be useful strategy to understand and improve the (hemi)cellulolytic capacity of the strains**.**

## Supplementary Information

ESM 1 (PDF 344 kb)
