# Supplementary material for: Optimisation of xylanases production by two Cellulomonas strains and their use for biomass deconstruction
Source: Appl Microbiol Biotechnol. 2021 May 21;105(11):4577–88. doi: 10.1007/s00253-021-11305-y (PMC8195749; doi:10.1007/s00253-021-11305-y)
Supplement: Supplementary file 1 — (PDF 344 kb) [file 253_2021_11305_MOESM1_ESM.pdf]

Supplementary Material

Applied Microbiology and Biotechnology

Optimization of xylanases production by two *Cellulomonas* strains and their use for biomass deconstruction

Ornella M Ontañon[1], Soma Bedő[2], Silvina Ghio[1], Mercedes M Garrido[1], Juliana Topalian[1], Dóra Jahola[2], Anikó Fehér[2], Maria Pia Valacco[3] Eleonora Campos[1*] and Csaba Fehér[2**]

[1]Instituto de Agrobiotecnología y Biología Molecular (IABIMO), Instituto Nacional de Tecnología Agropecuaria (INTA), Consejo Nacional de Investigaciones Científicas y Técnicas (CONICET), De los Reseros y N. Repetto s/n, Hurlingham B1686IGC, Buenos Aires, Argentina.

[2]Biorefinery Research Group, Department of Applied Biotechnology and Food Science, Budapest University of Technology and Economics (BUTE), Szent Gellért tér 4., H-1111, Budapest, Hungary.

[3]Centro de Estudios Químicos y Biológicos por Espectrometría de Masa (CEQUIBIEM-FCEN), Departamento de Química Biológica Facultad de Ciencias Exactas y Naturales Universidad de Buenos Aires (UBA-IQUIBICEN), Consejo Nacional de Investigaciones Científicas y Técnicas (CONICET)

* campos.eleonora@inta.gov.ar; ** feher.csaba@vbk.bme.hu

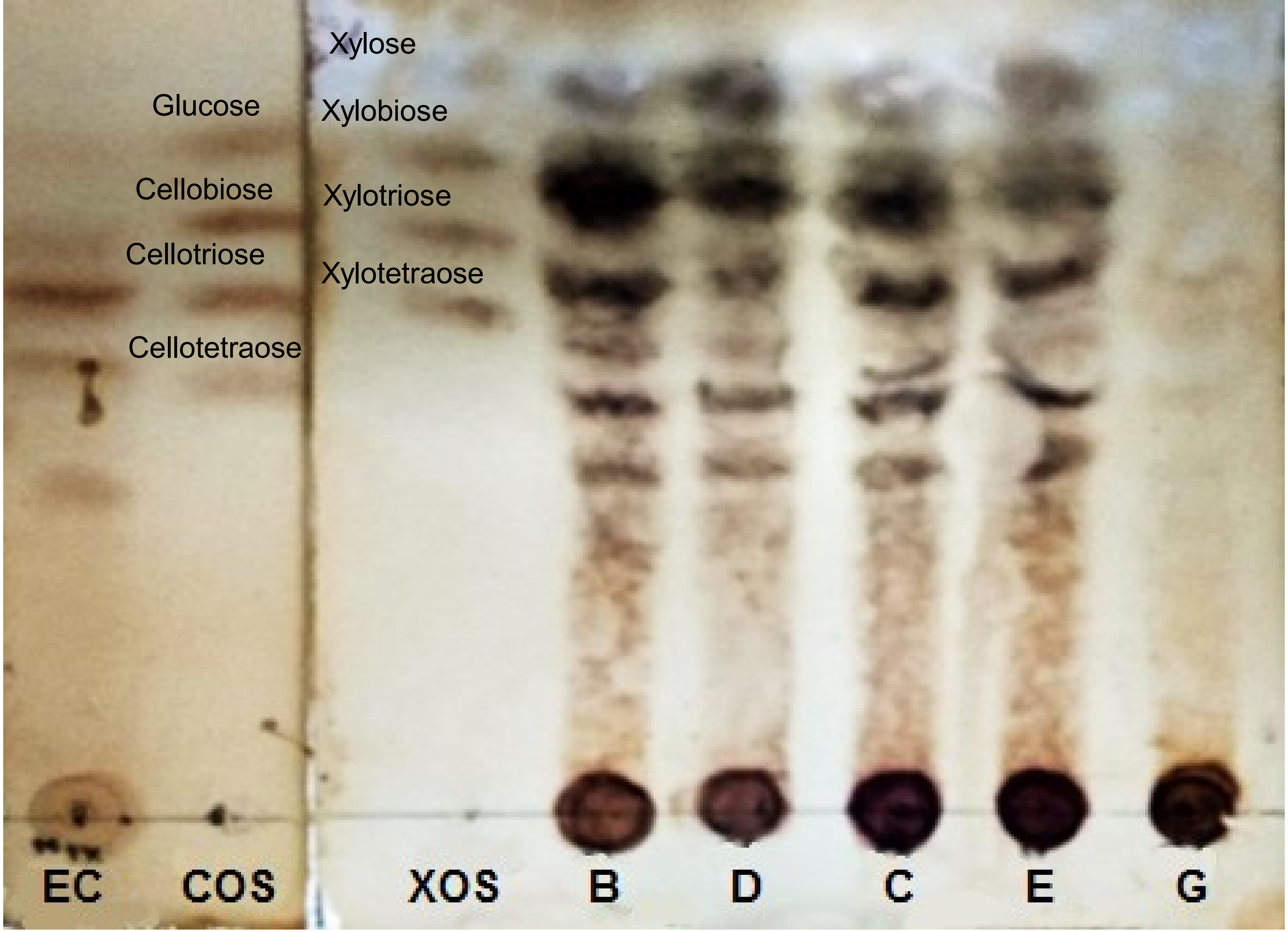

Fig. S1 TLC profiles of the hydrolysates obtained from extruded barley straw (EBS) by using different mixtures of extracellular (LEE) and intracellular (IE) fractions of *Cellulomonas* sp. B6 grown on wheat bran (WB). B and C correspond to 15 $U_{xylanase}$/mL of LEE and 7.5 $U_{xylanase}$/mL of LEE, respectively. D and E correspond to 15 $U_{xylanase}$/mL of LEE with 1.5 $U_{xylanase}$/mL of IE and 7.5 $U_{xylanase}$/mL of LEE with 0.75 $U_{xylanase}$/mL of IE, respectively. G: Substrate control. EC: Enzymatic extract control. COS: Cellooligosaccharide standards. XOS: Xylooligosaccharide standards.
